# Large language models (LLMs) as agents for augmented democracy

**DOI:** 10.1098/rsta.2024.0100

**Published:** 2024-11-13

**Authors:** Jairo F. Gudiño, Umberto Grandi, César Hidalgo

**Affiliations:** ^1^ Center for Collective Learning, University of Toulouse & Corvinus University of Budapest, Toulouse, France; ^2^ IRIT, Université Toulouse Capitole, Toulouse, France; ^3^ Toulouse School of Economics, Université Toulouse Capitole, Toulouse, France

**Keywords:** digital democracy, algorithmic democracy, artificial intelligence, digital twins, direct democracy, natural language processing

## Abstract

We explore an augmented democracy system built on off-the-shelf large language models (LLMs) fine-tuned to augment data on citizens’ preferences elicited over policies extracted from the government programmes of the two main candidates of Brazil’s 2022 presidential election. We use a train-test cross-validation set-up to estimate the accuracy with which the LLMs predict both: a subject’s individual political choices and the aggregate preferences of the full sample of participants. At the individual level, we find that LLMs predict out of sample preferences more accurately than a ‘bundle rule’, which would assume that citizens always vote for the proposals of the candidate aligned with their self-reported political orientation. At the population level, we show that a probabilistic sample augmented by an LLM provides a more accurate estimate of the aggregate preferences of a population than the non-augmented probabilistic sample alone. Together, these results indicate that policy preference data augmented using LLMs can capture nuances that transcend party lines and represents a promising avenue of research for data augmentation.

This article is part of the theme issue ‘Co-creating the future: participatory cities and digital governance’.

## 1. Introduction

In principle, democracy is a government *of*, *for* and *by* the people. In practice, however, democracies are constrained by trade-offs between the frequency, scope and means of deliberation and participation. Today, many democracies rely on the use of intermediaries between the people and their sovereign power. This need for intermediation, however, has repeatedly come into question with improvements in communication technologies.

Back in the 1960s, Joseph Licklider, an American psychologist and computer scientist, suggested that a ‘man-computer symbiosis’ of ‘cooperative interaction’ could perform ‘collaborative decision-making tasks’ better than either part alone [1,2]. More recently, this need for intermediation was questioned first, by the proponents of e-democracy and web 2.0. solutions [3–6], and more recently, by work exploring the use of artificial intelligence (AI) to augment democratic participation [7–11].

In this paper, we explore the use of large language models (LLMs) as a method to create software agents that can power augmented democracy systems. That is, we explore the use of LLMs to train personalized digital twins that can act as intermediaries or assistants designed to augment the participatory ability of each voter [7]. We build and test different versions of this system using off-the-shelf LLMs and data collected in an experiment involving the collaborative construction of a government programme during the 2022 Brazilian presidential election [12]. In that experiment, volunteers were asked to select among 67 policies extracted from the government programmes of Brazil’s two main presidential candidates: Luis Inácio ‘Lula’ da Silva and Jair Bolsonaro. These volunteers selected their preferred proposals out of randomly chosen pairs of proposals, providing nuanced information about their policy preferences. Volunteers were also asked to self-report a variety of demographic characteristics, including sex, political orientation, location and age.

Here, we use these open data [12] to fine-tune six popular off-the-shelf LLMs (Llama−2 7B, LLaMA−3 8B, GPT 3.5 Turbo, Mistral 7B, Falcon 7B, Gemma 7B) and explore the potential and limitations of using them to build software agents for augmented democracy. We compare the accuracy of these LLMs to the one obtained using a bundle rule assuming that citizens with a self-reported political orientation (e.g. left/right) always select the proposals found in the government programme of the candidate sharing their political orientation. We find that LLM predictions tend to be more accurate than the predictions obtained using a bundle rule. This suggests that fine-tuned LLMs are able to capture nuances in a citizen’s preferences that go beyond what can be predicted only from party lines. At the aggregate level, we study the ability of a probabilistic sample augmented using LLMs to predict the aggregate preferences of the population. We find that probabilistic samples augmented using LLMs provide more accurate estimates of population-level aggregates of preferences than probabilistic samples alone. Finally, we introduce a diagram explaining different types of augmentation that can be applied to preference data. These findings advance our understanding of the use of software agents to create systems of augmented democracy.

### (a) Democracy and technology

Democracy is an institution that has long been bound and shaped by communication technologies. It is hard to think about the rise of modern democracies without the printing press [13–15], just like it is hard to understand the democratic practices of the twentieth century without acknowledging the role played by newspapers, radio and television. In the last 30 years, the communication landscape changed once again with the growth of the Internet, a technology that has also affected the practice of modern democracy [16–18]. In this paper, our goal is not to study the impact of communication technologies on modern forms of democracy, but to explore the use of a new technology (LLMs) as a method to construct agents to augment civic participation.

In brief, augmented democracy is the idea of using software agents to explore fine-grained forms of civic participation. These are forms that interpolate between representative and direct forms of democracy, where individuals not only choose among representatives, but can directly indicate their preferences on policy proposals [7–10].

In a representative democracy, parties or candidates represent bundles of proposals. Citizens are required to choose among competing bundles. This bundling is designed to help decrease the cognitive burden of citizens by reducing the number of options. Yet, while there are incentives for parties and politicians to adapt their bundles to their constituents, there is no guarantee that the bundles they choose are optimal at satisfying the preferences of all citizens. By using software agents, as an alternative way to alleviate the cognitive burden of citizens, augmented democracy provides an opportunity to explore forms of civic participation that unbundle policy proposals. For instance, by allowing each citizen to train a personalized software agent that can work for them as their representative. Augmented democracy systems, therefore, could be used to estimate personalized bundles for each citizen and explore unbundled forms of democracy. That is, the use of software agents is an invitation to explore the creation of collective decision-making systems that could be hard to build in the absence of this technology.

LLMs provide an interesting opportunity for the design of augmented democracy systems as they satisfy a few important conditions: (i) they are easy to use, (ii) they operate directly over natural languages and (iii) they are part of a competitive market populated by a wide variety of suppliers. LLMs language abilities make them an interesting choice for the creation of systems interacting directly with policy proposals and citizen preferences expressed as text. In fact, LLMs are good at simulating human responses in opinion polls [19–22] or predicting votes in binary elections [19]. Also, since LLMs are trained on large bodies of text, they are likely to encode information on the policy preferences of a wide variety of people, which can be potentially extracted with the right prompting (e.g. by using backstories to create personas [19]) or fine-tuning. From the perspective of industrial organization, today LLMs are part of a competitive global market including hundreds of options (the latest Open LLM leaderboard on Hugging Face lists hundreds of LLMs [23]). This competitive market is an important institution, as the ability of people to switch among LLM providers reduces the risk of manipulation and/or capture. Yet, there are also important caveats that we need to consider when exploring the augmented democracy potential of LLMs.

LLMs are known to exhibit biases and limitations [24–27], which could set a ceiling on their ability to represent people with different political views and opinions, especially those groups that do not participate in the creation of potential training data (e.g. remote and offline indigenous communities). Also, LLMs are a powerful technology following a wide variety of governance models that can lead to different forms of manipulation and capture. Some LLMs, such as those released by OpenAI, are developed as proprietary models in ways that are untransparent about their source of training data. Other LLMs, such as LLaMA, are developed in an open-source model but still rely heavily on the support of researchers employed in a private sector organization. Other LLMs such as Mistral, rely on venture funding, while others, such as Falcon, depend directly on government support, in this case the government of Abu Dhabi. All of these governance models are not immune to potential capture or manipulation. Finally, LLMs exhibit poor logical skills in some tasks (they have been notoriously bad at mathematics and struggle with spatial reasoning), which could be problematic in decision-making tasks requiring these skills. These limitations need to be taken seriously before any real-world implementation of an augmented democracy system.

Our work also complements several studies exploring different aspects of digital and/or augmented democracy. The technical literature on augmented democracy includes efforts focused on cryptographic solutions for privacy and verification [28,29] as well as work on data augmentation using matrix completion techniques [29] or LLMs, as shown in recent work on participatory budgets [8] or direct democracy in Switzerland [30]. This technical work has also explored the creation of deliberative forms of augmented democracy—where LLMs respond to each other—by creating conversational social media agents [31] or by using LLMs to simulate the responses of citizens with different political views [19,32]. The idea of augmented democracy also builds on recent work showing that LLMs can be used to simulate human participants in surveys [19–22], which has shown, for instance, that LLMs provide similar moral judgements to humans [20] and can be used to construct fine-grained personas and predict their electoral behaviour [19]. On the philosophical side, the exploration of LLMs has focused mostly on the critical and ethical comparison of different forms of digital democracy, including augmented democracy [9,10,33,34,34,35].

Finally, it is worth noting that augmented democracy is an idea that stems naturally from recent advances in crowdsourcing and AI [12,36–43]. The set-up used in this study closely resembles work in urban planning, where a paired comparison system was used to collect information on people’s preferences for streetscapes [39] and then used to train machine learning models that augmented that data to produce fine-grained evaluative maps of cities [44–46]. In this paper, we follow a similar set-up, but instead of using information about people’s preferences over streetscapes, we use information on their preferences across policy proposals. We use this set-up to introduce different modes of augmentation and to develop benchmarks for both individual and aggregate preferences. These findings contribute to our understanding of the use of LLMs as a method to create agents for augmented democracy.

## 2. Results and methods

**Figure 1.**
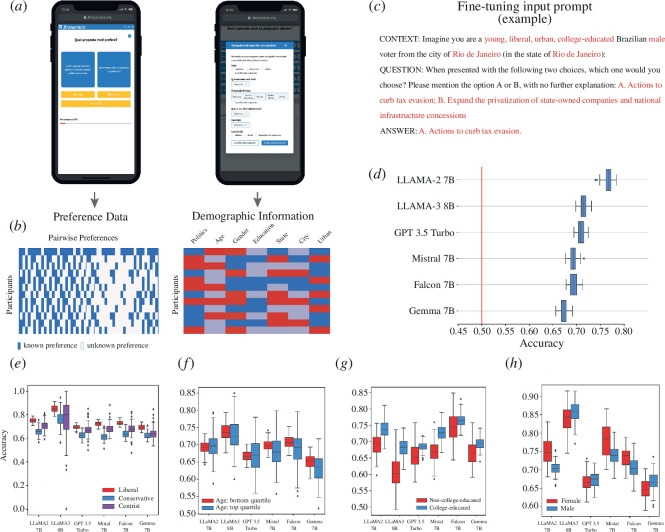
(*a*) Data were collected in brazucracia.org, a collaborative government programme builder deployed during the 2022 Brazilian presidential elections [12]. (*b*) Brazucracia data consisted of pairwise preference data and demographic data for each participant. (*c*) Example of the ‘mad-libs’ style prompt used to fine-tune the LLMs using a low-rank adaptation or LoRA process. (*d*) Accuracies obtained on the 50% test set of six LLMs. (*e–h*) Comparison of the accuracies obtained for samples with different self-reported demographic characteristics, by e political ideology, (*f*) age (older and younger quartiles), (*g*) education, and (*h*) sex. Samples in figures (*e–h*) were balanced by downsampling the variable with the largest representation in the data. Confidence intervals at 99% are calculated through bootstrapping with 100 iterations.

Figure 1*a,b* show the basic data we use to train and evaluate LLMs predictions. These are data collected in Brazucracia.org, an online participatory experiment conducted during Brazil’s 2022 presidential election [12]. In Brazucracia, participants were asked to select among pairs of proposals extracted from the government programmes of the two main candidates of the 2022 Brazilian election (see figure 1*a*). For instance, to prioritize among: *investing in clear and renewable energy* a proposal that was present in the programme of both Lula and Bolsonaro, or *strengthening of the subsided pharmacies programme* a proposal that was present only in the government programme of Lula.

During the process, participants were also invited to fill out a basic demographic survey (age, political ideology, rural/urban area, educational attainment, gender, age and geographic location), which we can use to connect their preferences to their stated demographic characteristics. The collection of these data was approved by TSE-IAST Review Board for Ethical Standards in Research, under the reference code 2022−07−001 and was made publicly available together with the publication of [12]. Our dataset consists of 8719 pairwise preferences elicited by 267 participants over a universe of 67 proposals. While these data are sparse (which is one of the conditions that motivates the need for augmented democracy), we estimate the test-retest reliability of the aggregate preferences of the full sample at 95.38% (see electronic supplementary material section B). This is consistent with previous work using paired comparison data in other contexts [39], since paired comparison rankings tend to converge at about 30 to 40 preferences per participant. More details about the dataset and data adequacy checks are presented in the electronic supplementary materials, appendices A and B and in Navarrete *et al*. [12].

We use these data to explore the ability of the LLMs to model the individual and collective preferences of this population of 267 participants. Going forward this population represents our statistical universe and we will refer to it as the full sample or complete sample of participants. That is, this study is not focused on estimating preferences for the general population of Brazil (as we lack the data to do so), but on understanding how samples from this universe of participants (e.g. a 20% random sample of only 53 participants) can be used to estimate the preferences of the entire universe of participants (the 267 participants).

First, we will explore the ability of LLMs to model individual preferences. That is, we will use a train-test cross-validation set-up to study the ability of the LLMs to predict the preferences of individuals withheld from the training data but available in the test data. Then, we will study the overall ability of the LLMs to reproduce the aggregate preferences observed in the data, by comparing the ability of pure and augmented random samples to reproduce the aggregate preferences of the full population of participants.

We begin by splitting our data randomly into a training set (50% of participants) and a test set (50% of participants). We use the training set to train LLMs using the following procedure (figure 1*c*):

First, we create prompts that encapsulate the demographic characteristics of each participant along with the pair of proposals they selected. For example, for a 26 year-old liberal male with a master’s degree living in Rio de Janeiro, who in *Brazucracia* selected the proposal: ‘*actions to curb tax evasion’* over the proposal ‘*expand the privatization of state-owned companies and national infrastructure concessions*’. We then feed the LLM such prompts as well as a prompt reversing the order of the proposals to compensate for the fact that LLMs exhibit biases depending on the order of the text [27] (figure 1*c*).

We use these prompts to fine-tune LLMs by retraining a fraction of their parameters (~12%) using a Low-Ranking adaptation (LoRA) technique, a method used in natural language processing (NLP) to improve the performance of pre-trained LLMs on predefined tasks [47]. LoRA is a Parameter-Efficient Fine Tuning (PEFT) method that works by freezing the model weights of the network and efficiently recalculating a fraction of the weights of the network to adapt it to the injected prompts. This allows the creation of customized LLMs with relatively little parameter adjustment. For reproducibility reasons, we fine-tune at temperature zero (but find that using different temperature parameters does not affect our results (see electronic supplementary material, Section H)). We use vLLM, a distributed serving engine for LLMs, to accelerate the generation of predictions [48].

Next, we test the ability of LLMs to predict the preferences of participants using six off-the-shelf LLMs: (i) GPT 3.5 Turbo [49], a model trained with proprietary architecture and data, (ii) LLaMA−2 7B, (iii) LLaMA−3 8B [50], (iv) Mistral 7B [51] and (v) Gemma 7B [52], four open-weight models trained on proprietary data, and (vi) Falcon 7B [53], an open-weight model developed by a government-sponsored research laboratory trained on public data. We also include a foundation model trained on public data (BERT SQuAD—BERT for Multiple Choice) [54] as a benchmark. We use this simpler model (of an older vintage and with fewer parameters) to test whether present day LLMs, which are oversized and more intensive on their use of computational resources [55], are needed for this augmentation task or if a simpler model would do. We do not present results for GPT−4 and Claude 3 as fine-tuning options were not yet publicly available at the time of writing this paper. Details of the fine-tuning process for each LLM are presented in the electronic supplementary material, appendix C.

### (a) Individual preferences

Figure 1*d* shows the percentage of times that a fine-tuned LLM trained on a random sample of 50% of the participants correctly predicts a pairwise choice from a participant in the test-set composed of the remaining 50%. A total of 95% confidence intervals are calculated through bootstrapping with 100 iterations. In this test, LLaMA−2 registers the highest accuracy (76.68% ± 0.0014) followed by GPT 3.5 Turbo (70.4% ± 0.0013), Mistral 7B (69.4% ± 0.0017) and Falcon 7B (69.3% ± 0.0014). BERT SQuAD does not perform better than random (50.83% ± 0.0015) (see the electronic supplementary material, appendix E), making it relatively unsuitable for this data augmentation task. In the electronic supplementary material, appendix E, we present results for fine-tuned LLMs trained on random samples of 5, 25 and 75%. In the electronic supplementary material, appendix F, we present results for the Kendall’s tau as an alternative metric of accuracy, which compares the number of congruent pairs (when the LLM and the citizen made the same choice) and incongruent pairs. Our data cannot be used to estimate an F1 statistic, since by construction, it couples true positives and true negatives, and false positives and false negatives, making F1 = Precision=Recall = Accuracy^1^.

Next, we explore how the accuracy of these models relates to the demographic characteristics of the population of participants. This will help us explore the potential biases of these models. For instance, we would like to determine if LLMs predict better the preferences of college-educated participants by comparing the accuracies obtained when predicting the preferences elicited by college and non-college-educated participants in the test set.

Because our data are unbalanced (e.g. we have more liberal than conservative participants), we retrain our models using balanced data subsamples generated by randomly selecting a smaller sample from the overrepresented subset. For instance, if our training data contain 70 individuals associated with characteristic A and 30 associated with characteristic B, we fine-tune a new model using the 30 individuals associated with B and a random sample of 30 individuals associated with A. We then generate predictions for individuals in the test set using these LLMs and compare the accuracies obtained for individuals associated with A and B.

Figure 1*e–h* show the accuracies obtained by the LLMs on subsets of participants with different self-reported political views (liberal/conservative) (figure 1*e*), age (younger and older quartiles) (figure 1*f*), education (college educated/non-college educated) (figure 1*g*) and sex (male/female) (figure 1*h*).

Across all six LLMs we find accuracies to be higher when predicting the preferences of liberal participants compared with conservatives and centrists (all *p*-values <0.01, see the electronic supplementary material, appendix I). When it comes to age, we find a slight but significant tendency to predict the preferences of younger participants more accurately in Mistral 7B, Falcon 7B and Gemma 7B. When it comes to education, we find that all LLMs predict the preferences of college educated participants more accurately than those of non-college educated participants (all *p*-values<0.01, see electronic supplementary material, appendix I). Finally, when we split our data by self-reported sex, we find a mixed bias. While LLaMA−2 7B, Mistral 7B and Falcon 7B are better at predicting the preferences of females, LLaMA−3 8B and Gemma 7B are more accurate at predicting the preferences of males. This contributes to the ongoing discussion of whether LLMs may overrepresent some segments of the population [56,57].

Next, we benchmark the accuracy of the LLMs against a bundle rule, representing the idea that voters in a representative democracy are required to choose among *bundles* of policies represented by parties or politicians. A bundle rule prediction consists of choosing proposals listed on the programme of the candidate that matches the political ideology of each participant. That is, predicting that a self-reported left-wing liberal chooses a proposal listed in Lula’s programme and a self-reported conservative chooses a proposal listed in Bolsonaro’s programme. We test the bundle rule benchmark using two different exercises.

First, we compare the accuracy of different LLMs disaggregated by the ideology of the participant and the ideology of the proposals. More concretely, we compare these accuracies using matrices that calculate the percentage of times that a preference is predicted correctly when: (i) a liberal chooses a policy listed in Lula’s programme (top-left) against a proposal listed in any other programme (but not in Lula’s), (ii) a liberal chooses a policy listed in Bolsonaro’s programme (top-right) against a proposal listed in any other programme (but not in Bolsonaro’s), (iii) a conservative chooses a policy listed in Lula’s programme (bottom-left) against a proposal listed in any other programme (but not in Lula’s), and (iv) a conservative chooses a policy listed in Bolsonaro’s programme (bottom-right) against a proposal listed in any other programme (but not in Bolsonaro’s). We exclude from the exercise cases in which the participant chooses between two proposals coming from the same candidate (e.g. a choice between two proposals present only in Lula’s programme) but explore this case later.

**Figure 2.**
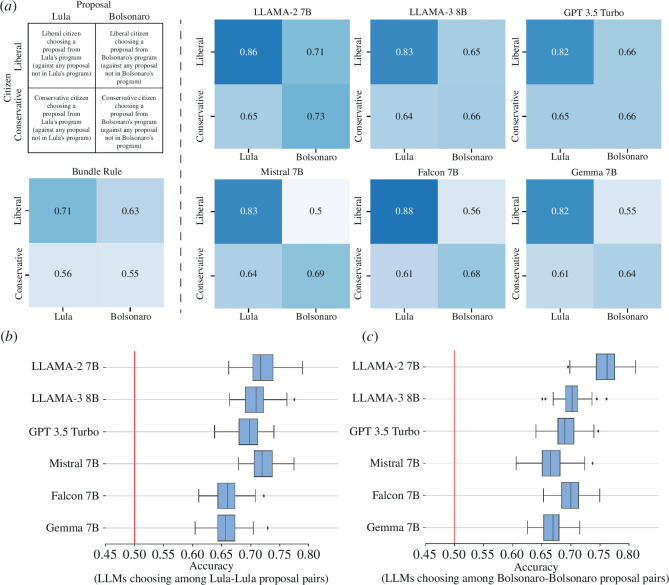
Comparison between LLMs and the bundle rule. (*a*) Accuracies obtained for the six different LLMs and the bundle rule when predicting pairwise preferences. (*b,c*) Accuracy of LLMs when predicting preferences between pairs of proposals found in the same programme, (*b*) Lula and (*c*) Bolsonaro.

Figure 2*a* shows that, across most cases, LLMs exhibit higher accuracies than the bundle rule (21 out of 24 cases or 87.5% of times). These differences are sometimes large. LLaMA−2 7B is 15 percentage points more accurate than the bundle rule at predicting which proposals from Lula are selected by self-identified left-wing participants. In fact, all LLMs get an accuracy of 80% or higher when predicting the policies of Lula’s programme selected by self-identified left-wing participants (compared with 71% for the bundle rule). Yet, we do find three exceptions, involving liberals choosing a policy listed in Bolsonaro’s programme for Mistral, Falcon and Gemma.

Our second approach, shown in figure 2*b,c*, considers predictions that cannot be made using the bundle rule. These are predictions involving a choice among proposals from the same candidate (e.g. preferences among two proposals from Lula’s programme). In this case, the LLMs still have significant predictive power for these cases (between 65 and 77%). Overall, we find that LoRA fine-tuned LLMs are better at predicting individual preferences than what we get from predictions based solely on self-reported political orientation.

### (b) Aggregate preferences

Next, we explore whether we can use these LLMs to improve our ability to estimate the aggregate preferences of the population starting from a random sample. As a benchmark, we estimate the accuracy with which a probabilistic sample of participants predicts the preferences of the full sample. That is, we ask if a probabilistic sample plus an LLM trained on that same sample is better at predicting the aggregate preferences of the full population than the probabilistic sample alone.

As a measure of aggregate preferences, we estimate the win rate of each policy proposal by aggregating the data obtained from the individual predictions. This is a Borda-inspired score that we can estimate for incomplete preference data. It is defined as the fraction of times a proposal is selected out of a pair. A win rate of one (or 100%) indicates that a proposal was always chosen over others and a win rate of zero indicates that a proposal was never chosen over others. The win rate is, therefore, a measure of the overall acceptance of a proposal among the population of participants.

Formally, let *w_ij_* be the number of times proposal *i* was selected over proposal *j*. Then the win rate *W_i_* of proposal *i* is defined as:


$$W_{i}=\;\frac{\sum_{j}w_{ij}}{\sum_{j}\left(w_{ij}+\;w_{ji}\right)}.$$

**Figure 3.**
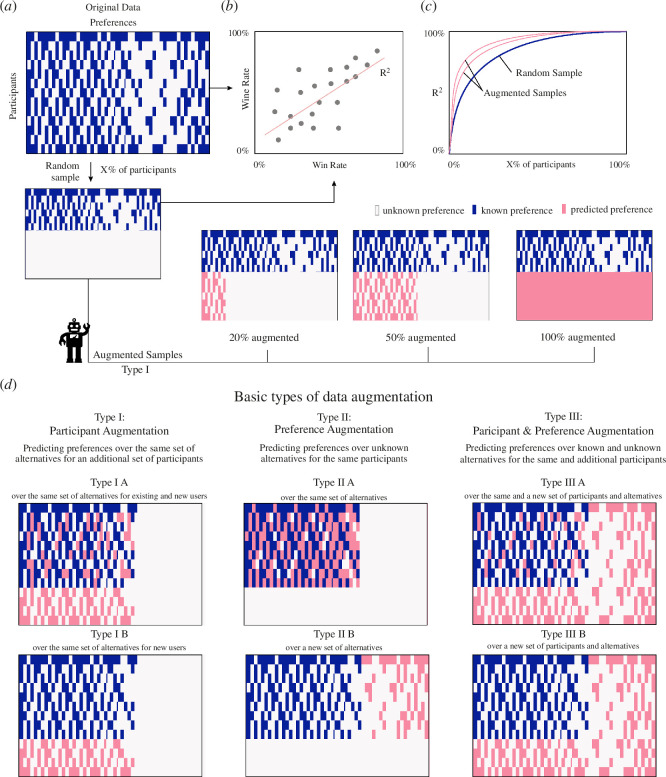
Augmentation and validation procedure. (*a*) We represent preference data using a matrix in which each row represents a participant and each column a preference over a pair of policy proposals. We can sample this data randomly, and augment that sample, to estimate the ability of the random and augmented samples to reproduce the data of the complete sample. (*b*) We assess the ability of a random or an augmented sample to reproduce the aggregate preferences of the full sample by comparing the win rates among the proposals and estimating the resulting *R*^2^ statistics. (*c*) We plot the *R*^2^ of the win rates comparing a random or an augmented sample of size x with the full sample as a way to estimate the accuracy with which that sample of size *x* represents the aggregate preferences of the full sample. (*d*) Different data augmentation procedures.

To estimate the ability of a random sample to represent the aggregate preferences of the full population we need to estimate the win rate *W_i_* of each proposal twice, once for the full sample (all 267 participants and all their elicited preferences) and another time using data from a random sample (figure 3*a*). We then compare the similarity between the estimated win rates by calculating the *R^2^* of their Pearson correlation (figure 3*b,c*). An *R^2^* = 100% indicates that the win rates obtained using the partial sample are identical than those obtained for the full population, meaning that the sample can reproduce the aggregate preferences of the larger population.

Next, we explain our data augmentation procedure (figure 3*d*). In principle there are multiple ways in which one could augment preference data. Figure 3*d* shows three types of data augmentation (types I to III). Type I involves augmentation of the participants in the sample, which involves predicting the preferences of additional participants based on external information about them, in our case, demographic characteristics (e.g. age, sex, education, etc.). Type II involves predicting additional preferences for the existing set of participants. This could involve predicting unelicited preferences over the same alternatives (type II A) or predicting preferences for new alternatives (type II B). In our case, type II B would include predictions over alternatives that were not part of the 67 proposals presented in Brazucracia. There is also a type II C that combines these two. Finally, type III involves predictions for both new participants and new alternatives (and is a combination of types I and II).

**Figure 4.**
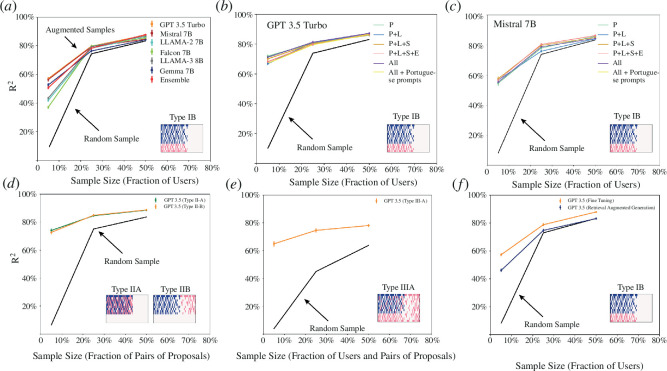
Comparing augmented and non-augmented random samples. We estimate the accuracy with which a sample of size *x*% is able to reproduce the aggregate ranking of preferences of the full population using the R^2^ statistic. (*a*) Comparison between a probabilistic sample of size x percentage of users and an augmented sample (type I B) generated with LLMs fine-tuned on the same data. (*b*) Same as (*a*) but using a different set of demographic characteristics to train each LLM (*p* = Political Ideology (liberal, conservative, centrist), L = Location (city and state), S = Sex (male, female), E = Education level (non-college educated, college educated). (*c*) Same as (*b*) but for Mistral 7B. (*d*) Type IIA and IIB augmentations using all demographic information. (*e*) Type IIIA augmentation using all demographic information. (*f*) Comparison of augmentation via fine-tuning and RAG for type IB augmentation.

Figure 4*a* shows a direct example of a type I B augmentation process using GPT 3.5 Turbo, LLaMA−2 7B, Falcon 7B, Mistral 7B, LLaMA−3 8B and Gemma 7B. The black dashed line shows the ability of a non-augmented random sample of 5, 25 and 50% of participants to reproduce the aggregate preferences of the full population and the coloured lines show the accuracy of these data augmented by each of these LLMs. We use this LLM to augment the data by predicting the elicited preferences of an extra 20% of the remaining population (e.g. 20% of the remaining 95% of the population in the case of the 5% sample). For the 5% random sample this provides a substantial boost, from about R^2^~30% to R^2^~75%, and even at a 25% random sample the boost provided by the LLMs is substantial (about 7 to 10 percentage points). We do not provide results for non-fine-tuned models as these models always choose the first alternative (e.g. 'A') at zero temperature. Finally, we present a consensus method (ensemble) that picks the prediction made by the majority of the LLMs but find that this does not work better than the single LLMs. These results show that an augmented sample can be better at reproducing the preferences of the full population of participants than a random sample alone.

Figure 4*b,c* repeat this procedure while varying the demographic characteristics used to train the LLMs (e.g. including or not information about a participant’s level of education) for both GPT 3.5 Turbo and Mistral 7B. Sensitivity tests for temperature are presented in the electronic supplementary material’, appendix H. Overall, we find that the accuracy of the augmented samples does not depend strongly on the demographic variables included or if we change the language of the training prompts and the consultation from English to Portuguese [58]. In the electronic supplementary material, appendix D, we present the prompt and description of features in Portuguese.

Figure 4*d* shows examples of type II A and B augmentation using GPT 3.5 Turbo. In this case, the augmentation process is even stronger than for type IB. We conjecture this is due to the fact that types IIA and IIB make better use of preference data, since each user is not characterized only by their demographic characteristics, such as in type IIB, but also by a sample of its elicited preferences. Figure 3*e* shows akin results for a type III A augmentation.

But what is the knowledge captured by these LLMs? Does it go beyond simple context? To explore these questions we compare two prediction methods implemented in GPT 3.5 Turbo: fine-tuning with LoRA [47] and Retrieval Augmented Generation (RAG) [59], a method based on providing additional context in the form of documents to the LLMs, in our case a document summarizing the proposals of the participant’s favourite candidate (Bolsonaro for conservatives; Lula for liberals). This comparison helps us evaluate whether the performance of the fine-tuned model comes from its ability to capture information that goes beyond the context made available through the RAG approach. We find that the LoRA fine-tuned predictions reproduce the preferences of the full population of participants better than RAG (figure 4*f*), especially for larger samples (25 and 50%). This validates the idea that the fine-tuning process can be used to create personalized software agents that provide a more accurate representation of an individual’s preferences than software agents created by providing context to the chat layer of the LLMs.

## 3. Discussion

Is the world ready to explore augmented forms of civic participation? In 2019, IE’s University Center for Governance and Change published a report where 2576 people from eight European countries responded to questions about digital technology [60]. According to García-Marzá & Calvo [61], this revealed a digital paradox, since 70% of respondents believed ‘digital transformation needs to be controlled to avoid’ its negative impacts in society, while a non-negligible proportion of the respondents (25%) indicated they be ‘in favour of letting an artificial intelligence make important decisions about the running of their country’. More recent studies also provide some credence to the idea that people may be willing to consider using AI in policy making. A study published in 2024 [62] reported that more than 60% of the people in their sample would approve of a decision-making model in which AI has 25% of the decision-making weight and politicians 75%. The same study showed that people also are more willing to give some weight to an AI in technical tasks, but still would prefer to defer to experts in that case.

These numbers provide some food for thought. On the one hand, we live in a world that is anxious about the societal implications of digital technology. On the other hand, there seems to be an important number of citizens that are willing to give some civic power to AI. But are those willing to give AI a chance in theory willing to do so in practice? The evidence so far points to the contrary. A first wave of efforts to create centralized AI representatives, such as SAM in New Zealand [63], Alisa in Russia [64], Leader Lars in Denmark [65], ION in Romania [66] and AI Mayor in Japan [67], gathered more media support than success at the ballot box. The case of AI Mayor in Tama City, a 150 000 people suburb of Tokyo, is particularly interesting, since the support for AI Mayor decreased instead of increased in subsequent elections. In its first appearance, in 2018, it made it to the second round after receiving 4013 votes. In a more recent election in 2022 the AI Mayor received only 516.

Against this backdrop of efforts, augmented democracy remains still a relatively unexplored idea [8,30]. It is also different from the creation of AI politicians, as it does not involve the creation of a single AI representative designed to ‘listen to everyone’, but an ensemble of AI agents, each controlled by its own human: citizens can create individual profiles that can be personalized according to their own characteristics, preferences and habits and these autonomous agents can potentially vote on their behalf.

In this paper, we contributed to the early exploration of augmented democracy systems by fine-tuning six off-the-shelf LLMs and studying their ability to anticipate organically collected fine-grained political preference data collected during the 2022 Brazilian presidential election [12]. We found that LLMs predict pairwise preferences with higher accuracies for participants that self-reported as younger, liberal and more educated. We then explored the ability of LLMs to augment participation data in an exercise in which we used a sample of our data to predict the aggregate preferences of the full population of participants. This exercise showed that the LLMs provide an effective augmentation for small sample sizes (less than 30%), resulting in estimates of the aggregate preferences of a population that are more accurate than those obtained from non-augmented random samples. We also found the LLMs to be more accurate than a bundling rule (assuming people always select the policies of their preferred party or politician) suggesting that LLMs capture information that is more nuanced than a simple left–right wing divide.

Yet, despite these findings, this paper still leaves many unexplored questions. On the one hand, we use LLMs only in a context of preference aggregation, where the idea of using AI to explore augmented forms of multi-agent consensus is also a promising avenue of research [68–70]. Also, we do not explore how traditional forms of multi-agent consensus might change in the context of preferences being elicited in an augmented democracy system. On the other hand, future research must explore whether the performance of LLMs is contingent on the design of the platform. Our results are based on data from Brazucracia, a platform that differs from the design of other Voting Advice Applications (or VAAs, like Wahl-O-Mat or Elyze) in an important aspect. In Brazucracia, the objective is to rank-sort the most relevant proposals for citizens whose preferences might not align perfectly with a specific political party, instead of recommending a party or politician to the user. We also do not use multi-agent systems, which in some cases have been shown to be more accurate than single agent LLMs [71,72]. Finally, our exploration of LLMs was far from comprehensive, and did not include some of the latest models (e.g. GPT−4 and Claude 3) or multiple variations in the prompts used to train and query the LLMs. But these are only some of the limitations of our work.

There are also important limitations involving the representation of both preferences and participants. The idea of augmented democracy is based on the construction of digital twins, which in the case of this paper, was constructed using a minimalistic representation of each agent (a relatively short vector or demographic characteristics and pairwise preferences). This is a far cry from the state-of-the-art in the creation of digital twins, such as the recent digital twin demo released by LinkedIn founder’s Reid Hoffman [73]. The demo involved a video interview in which Hoffman interviewed and was interviewed by his digital twin, which was trained among other things on many of his books. Certainly, digital twins could be made more accurate with more and better data, but the differential availability of that data adds to the challenge of political representation, since the production of the data needed to train AI systems (e.g. text, video) is uneven among the population.

Today, LLMs are not ready for deployment in fully fledged augmented democracy systems but provide an interesting avenue of research in that direction. This research needs to address key concerns.

First, the data used to pre-train many of these LLMs (e.g. GPT 3.5 Turbo and Mistral 7B) are proprietary and could be open to manipulation. The sensitive nature of augmented democracy system requires us to think deeply about the open-source code and open data rules needed to develop these systems in a transparent manner.

Second, LLMs can generate ambiguous predictions that sometimes depend on the order in which options are presented (A versus B, or B versus A). In some cases, this consistency can be lower than 70% (see the electronic supplementary material, section G).

Third, LLMs exhibit biases. In this paper, we found LLMs to be more accurate at predicting participants that self-reported as liberal and more educated, and some LLMs tended to exhibit a bias in favour of females. This talks to the long literature in NLP and LLMs discussing gender bias [74–77]. Yet, our results are somehow different since that literature focuses largely on how LLMs use language, for instance, to disambiguate words such as *doctor* and *nurse* (e.g. assume doctors are males and nurses are females). In this paper, we are not using LLMs to disambiguate gender but to predict their civic preferences. Thus, we cannot assume that the same bias (e.g. favouring males) operates in this context. Still, the literature provides some important points of comparison.

A recent paper by Argyle *et al*. [19] uses LLMs (GPT3) to simulate human responses in surveys. Argyle *et al*. find that LLMs given personal backstories constructed to represent a particular demographic are able *to accurately emulate response distributions from a wide variety of human subgroups*. In their study, Argyle *et al*. compare voting predictions made by these LLMs by gender, finding the LLMs to be slightly more accurate at predicting the preference of females (about one percentage point). Santurkar *et al*.[21] is another interesting study that also uses LLMs to explore human opinions in a question/answering set-up. In their case they find that LLMs tend to more accurately reproduce the preferences of young, low-income, moderates with less than high-school education. Santurkar *et al*. [21] reports that the LLMs used in their study are more accurate at predicting the preferences of males, but these differences are also small (about one percentage point or less).

Fourth, we lack a good framework of explainability, since we do not have a thorough understanding of *why* LLMs choose one proposal over another.

These are a few of the many limitations involved in an approach like the one we present here, meaning that much work remains to be done. Aside from addressing the previously mentioned shortcomings, it would be interesting to analyse cases of data augmentation involving other forms of data collection, such as those used in VAAs. Another interesting avenue of research is that of aligning LLMs toward different political viewpoints and then summarizing these views using other LLMs, as shown in the work of [78]. Furthermore, it would be interesting to explore if augmentation is improved by RAFT [79], a recent proposal based on combining additional external knowledge (RAG) and fine-tuning. Ultimately, these advancements will require further interdisciplinary research as the potential impact of AI as a tool for augmenting democracy is rather uncharted territory. We hope these findings contribute to stimulate and organize that exploration.

## Data Availability

Data collected from Brazucracia.org for this study are available in Harvard Dataverse [80]. Replication codes for figures, training files used in fine-tuning and resulting files are available at https://github.com/CenterForCollectiveLearning. While public access to open-source fine-tuned LLMs is supported, access to fine-tuned GPT-3.5 Turbo models remains restricted according to the OpenAI website. Interested readers may request a short-term OpenAI API key to replicate the exact results. Supplementary material is available online [81].

## Footnotes

^1^
We do not present an F1 score is because our data structurally couples true positives (TP) and true negatives (TN) and false positives (FP) and negatives (FN). Consider an LLM that is asked to make a choice between options A and B in a dataset where we know the user chose option A. If the LLM chooses A we have a true positive for A and a true negative for B. If the LLM chooses B we have a false negative for A and a false positive for B. Hence precision: Precision = TP/(TP+FP) and Recall = TP/(TP+FN) are the same Since FP=FN (Precision=Recall). Thus, F1 = 2TP/(2TP+2FP) = TP/(TP+FP) = Precision = Recall = Accuracy.
